# Enamel Thickness before and after Orthodontic Treatment Analysed in Optical Coherence Tomography

**DOI:** 10.1155/2017/8390575

**Published:** 2017-01-24

**Authors:** Julia Seeliger, Monika Machoy, Robert Koprowski, Krzysztof Safranow, Tomasz Gedrange, Krzysztof Woźniak

**Affiliations:** ^1^Division of Orthodontics, Technical University Dresden, Dresden, Germany; ^2^Division of Orthodontics, Pomeranian Medical University, Szczecin, Poland; ^3^Department of Biomedical Computer Systems, University of Silesia, Faculty of Computer Science and Materials Science, Institute of Computer Science, Katowice, Poland; ^4^Department of Biochemistry and Medical Chemistry, Pomeranian Medical University, Szczecin, Poland

## Abstract

Despite the continuous development of materials and techniques of adhesive bonding, the basic procedure remains relatively constant. The technique is based on three components: etching substance, adhesive system, and composite material. The use of etchants during bonding orthodontic brackets carries the risk of damage to the enamel. Therefore, the article examines the effect of the manner of enamel etching on its thickness before and after orthodontic treatment. The study was carried out in vitro on a group of 80 teeth. It was divided into two subgroups of 40 teeth each. The procedure of enamel etching was performed under laboratory conditions. In the first subgroup, the classic method of enamel etching and the fifth-generation bonding system were used. In the second subgroup, the seventh-generation (self-etching) bonding system was used. In both groups, metal orthodontic brackets were fixed and the enamel was cleaned with a cutter fixed on the micromotor after their removal. Before and after the treatment, two-dimensional optical coherence tomography scans were performed. The enamel thickness was assessed on the two-dimensional scans. The average enamel thickness in both subgroups was not statistically significant.

## 1. Introduction

Fixed braces are controversial because of the way they are attached to the tooth surface and a potentially devastating effect on the tooth enamel. Therefore, it becomes necessary to conduct research on the state of the tooth enamel after orthodontic treatment, depending on the used techniques and materials for fixing brackets. Research on this subject enables developing treatment procedures optimal for the enamel quality.

In clinical orthodontics, adhesive systems, whose structure is based on resin composites merging with enamel through an etching process, are most often used for bonding brackets. The purpose of etching is partial dissolution of the enamel minerals, which allows mechanical retaining of the orthodontic resin in the tissue pores created by an inorganic acid. It significantly increases the roughness of the enamel, enhancing the risk of plaque and sediments around the bracket, and reduces the hardness of the tissue and its resistance to external factors. Due to the effect of dissolving the enamel, it is very important to perform this procedure cautiously and skillfully and study possible alternatives to the above technique.

In the classic etching method, a relatively strong acid is used, which is usually a 35–40% solution of orthophosphoric acid. The solution is applied to the clean enamel surface during 15–30 seconds and then rinsed, and the enamel surface is dried using a strong air flow. The studies of Retief [1], Arakawa et al. [2], Asmussen [3], and Charbeneau Voss and Charbeneau [4] on the procedure of direct decalcification, evaluated using an optical microscope, showed a penetration depth of the etching acid into the tissue ranging from 5 to 50 *μ*m. In the course of the development of adhesive technology in dentistry, aiming to minimize the steps of attaching hooks, three separate elements were combined into two, combining the properties of the etchant and adhesive system [5–8]. Self-etching primers (SEP), owing to the presence of an acid primer, allow for the exclusion of the etchant [9, 10]. In the light of published studies [11], both ways of enamel etching show a similar pattern of the enamel porosity. The etching primer has a more classic pattern of etching [12–24] while maintaining an adequate, optimal bonding strength [8], similar to the strength generated using the classic method of enamel etching [15, 16].

The SEP bond strength, according to researches, ranges from 20 to 30 MPa [15], which shows a similar range of forces to the classical acid etching [16]. The big advantage of the system is the primers penetration on the entire depth of the generated pores of the enamel, which provides predictable, extremely durable mechanical fixation [17]. In the course of studies it has demonstrated that the extent of penetration of the glue is smaller using the same etching system than the normal etching. But this is not a disadvantage, because the greater hook in the enamel resin is, the greater risk of damage during removal of the debonding exists.

Many studies have shown that the extent of penetration of the glue is smaller using the self-etching primer than in the case of normal etching. However, this is not a disadvantage since the larger the resin hooks in the enamel, the greater the risk of its damage while debonding [18]. Considering this hypothesis, the presented article examined the effect of the method of enamel etching on its thickness before and after orthodontic treatment.

## 2. Material Methods

The study was carried out in vitro. The material comprised 80 teeth, divided into two groups of 40 teeth each. In the first test group, the orthodontic brackets were attached to the tooth surface using the fifth-generation adhesive system that uses the classic method of enamel etching with orthophosphoric acid. In the second group, the orthodontic brackets were attached to the tooth surface using the self-etching primer (seventh-generation system). In each group steel orthodontic brackets were used.

The experiment was carried out on the premolars, extracted for orthodontic and periodontal reasons. The exclusion criterion was defined by the following conditions: the presence of developmental defects of enamel, that is, hypoplasia, turbidity or discoloration, caries, and fillings on the vestibular surface.

The teeth qualified for research were stored for 30 days in demineralised water, with a crystal of thymol (0.1%) at room temperature.

Before fastening orthodontic brackets, the tooth surface was cleaned using a polisher (TopDental, Poland) with fluoride-free toothpaste Pressage (Shofu Inc., Japan) designed to prepare the enamel before fastening orthodontic brackets. Then, the tooth was washed with distilled water and dried with compressed air for 15 seconds. For fastening orthodontic brackets, an orthodontic composite material Transbond™ XT Light Cure Adhesive (3M Unitek, USA) was used, which requires the prior preparation of the enamel surface.

In the first group, the vestibular surface of the tooth was etched for 30 seconds with a 37% solution of phosphoric acid, Blue-Etch (CERKAMED, Poland), rinsed with distilled water for 15 seconds and dried using compressed air. The adhesive system OptiBond Plus Solo (Kerr, USA) was rubbed with an applicator into the etched enamel surface for 15 seconds; then the surface was dried under a gentle stream of air for 3 seconds and cured with a halogen lamp of the light intensity of 750 mW/cm^2^ for 20 seconds. The orthodontic composite material Transbond XT Light Cure Adhesive was applied to the bracket surface. The hook was pressed against the enamel surface with commonly used tweezers. The orthodontic hook was placed in the middle of the mesial-distal axis of the tooth, moving its centre 3.5 mm away from the edge of the occlusal surface. The distance was measured using an orthodontic positioner. After proper placement of the hook, the material was subjected to polymerization with a halogen lamp for 40 seconds.

In the second group, the self-etching adhesive system G-Bond (GC, USA) was used. The self-etching primer when applied to the tooth surface using an applicator was left for 10 seconds, and then the excess was removed via an air stream for 5 seconds. After this time, the system was polymerized with a halogen lamp of light intensity of 750 mW/cm^2^ for 20 seconds. The orthodontic composite material Transbond XT Light Cure Adhesive was applied to the surface of the hook. The orthodontic hook was placed onto the tooth surface using the above-described method.

The teeth with the fixed orthodontic brackets were stored in demineralised water at room temperature for 24 hours. After this time, the hooks were removed mechanically with pliers ix827 (Ixion Instruments, USA) designed for removing all types of hooks.

Residues of the adhesive material were removed from the enamel surface using a cemented carbide milling cutter H390.204 AGK (Komet URPOL, Poland) which has 8 notches, the size of 314.018, the length of 3.6 mm, and a diameter of 1/10 mm.

The enamel was processed with the use of a micromotor commonly mounted to a dental unit at a speed of 40000 revolutions/min with water cooling and pressure force of 1.0 N. The force was measured on a test stand consisting of scales, on which the processed tooth was placed.

The procedure of cleaning the enamel was considered to be finished on the basis of the naked-eye examination and by touching with the stylet 23 in the dental unit light. The assessment criterion was the smoothness of the tooth surface and the absence of the composite material residues.

### 2.1. Performance of Tooth Scans Using 3D-OCT

The area of the test teeth was imaged with a 3D-OCT camera (Topcon, USA, Figure 2) three times:

T0: imaging of the tooth surface before installing orthodontic brackets,
T1: imaging of the tooth surface after mechanical processing.

Each time, two-dimensional scans were performed allowing for a clear illustration of the enamel damage in a vertical plane. The procedure enabled showing the entire surface of the tissue and performing the subsequent comparative analysis of changes in its structure. The 3D-OCT device (Topcon, USA) in addition to CT has a coupled digital camera with a resolution of 16.2 Mpix, which provides highly accurate images of the test area with twentyfold zoom without losing image quality.

The technology of Fourier Domain OCT (S-OCT), which uses spectral analysis, provides very quick scanning (27000 A-scans/sec) and a high axial resolution of 5 *μ*m and a horizontal resolution of 20 *μ*m. The use of a pulsed light source, which is a superelectroluminescent diode (SLED) in the OCT, allows for better detection of low-contrast centres. The wavelength is 840 nm; the half-width is 50 nm. The 3D-OCT-2000 has a scanning range of 6 × 6 mm horizontally and 2.3 mm into the tissue. It is a device designed for ophthalmic diagnostics, whose system enables virtual segmenting of the retina into layers allowing for the assessment of the photoreceptors and pigment epithelium. The wavelength of 840 nm and the depth of penetration into the tissue also allow for imaging of the tooth enamel tissue through its entire thickness.

It was possible to obtain accurate scans of the surface and enamel structure of teeth with due repeatability during three examinations owing to a special matrix made for each tooth. The matrix allowed for repeatable tooth positioning in the frontal, sagittal, and horizontal plane relative to the optical axis of the OCT. The matrix was made of the c-silicone Zetalabor hard 85 Shore A (Zhermack, Italy), on the basis of the tooth impression in the long axis so that the vestibular surface of the crown remained above the silicone. The support for the silicone was a mould with fixed attachment with respect to the optical axis of the OCT.

**Figure 1:**
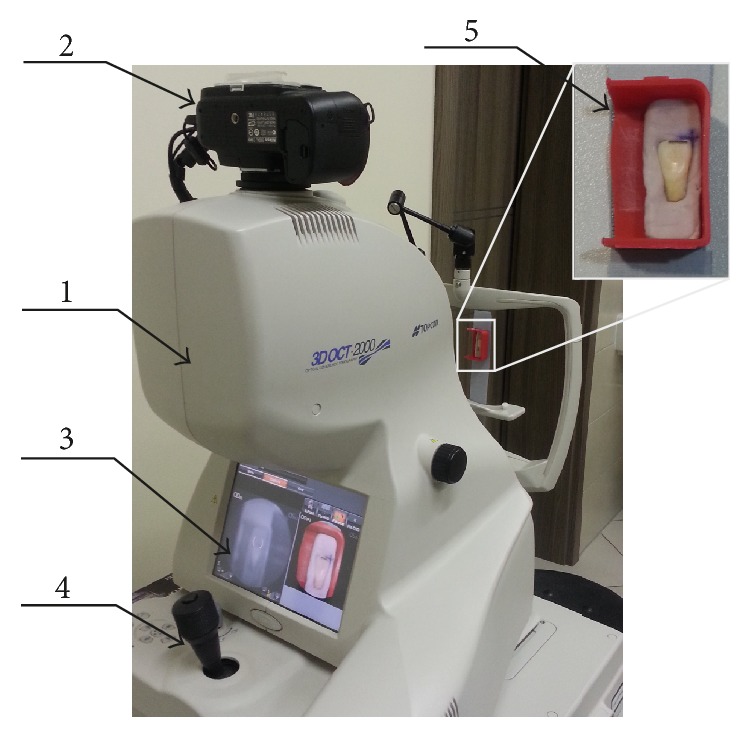
Image showing the method of acquisition of OCT images of the teeth. The following items are depicted: 1, OCT tomograph, 2, digital camera for taking images in visible light, 3, screen of the tomograph, 4, joystick enabling changing object position, and 5, method of attachment of the tooth in the device.

**Figure 2:**
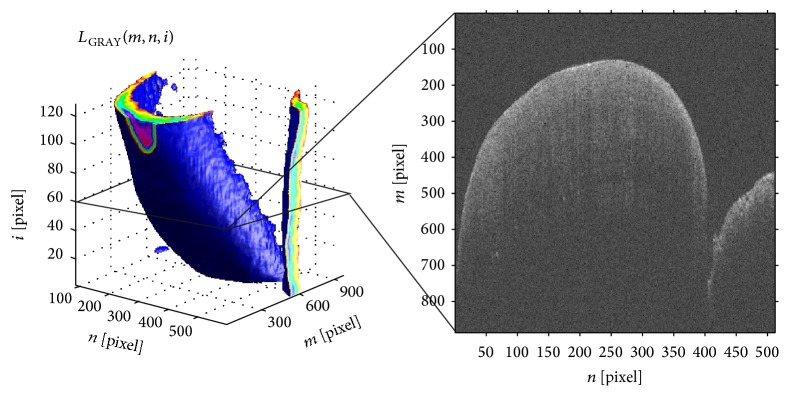
Reconstruction of a sequence of images *L*_GRAY_(*m*, *n*, *i*) for *M* × *N* × *I* = 884 × 512 × 128 pixels. The image shows the result of reconstruction of an image sequence with a sample B-scan obtained for *i* = 60. The analysis aims at automatic determination of the edge on a sequence of OCT images (B-scans) in order to determine enamel thickness.

The obtained OCT scans were subjected to an expert IT analysis. Image preprocessing involved automatic reading of the order of OCT images from the source file with the extension *∗*.fds allowing for the development of matrices of individual images.  Figure 1 shows the method of acquisition of OCT images of the teeth. Figure 2 depicts the reconstruction of the sequence of the OCT images. IT analysis, which was performed owing to a specially developed algorithm, was accurately described and published [25].

The results obtained in the study were statistically analysed. The Shapiro-Wilk test was used to verify the hypothesis of normality of variable distribution.

To verify the hypothesis of the existence and nonexistence of differences between the mean values for the independent variables, the median test and the Mann–Whitney *U* test were used. To verify the hypothesis of the existence or nonexistence of differences between the mean values for the dependent variables, the Friedman two-way analysis of variance and Wilcoxon matched-pairs signed-ranks test were used.

In order to assess the correlation between saccadic and qualitative variables, the chi-square test of independence, the chi-square test of independence with Yates' correction, and the Fisher's exact test were used. Maxwell's general principle was followed when using this type of tests.

The diversity of many variables in the categories determined by qualitative factors was analysed using models of univariate analysis of variance, ANOVA/ANCOVA. When verifying all hypotheses, the level of significance was *p* = 0.05.

## 3. Results

The results of the statistical univariate analysis, evaluating the difference in the enamel thickness after orthodontic treatment depending on the adhesive system, have not confirmed the relationship between the thickness of the enamel tissue after completed orthodontic treatment and the adhesive system. To carry out the above analysis, average, minimum, and maximum values of the tissue thickness after treatment as well as average, minimum, and maximum differences between the initial and final enamel thickness were used. In the case of the fifth-generation system, the tissue thickness after treatment amounted to 472,75 *μ*m, 128,18 *μ*m, and 10093,62 *μ*m, respectively, and the differences in thickness were 96,53 *μ*m, 55,71 *μ*m, and 432,69 *μ*m. When the seventh-generation system was used, the tissue thickness after treatment amounted to 469,03 *μ*m, 132,26 *μ*m, and 1103,84 *μ*m respectively, and the differences amounted to 90,93 *μ*m, 50,15 *μ*m, and 402,10 *μ*m. Among these measurements the most reliable was again the difference in the average enamel thickness (Dif_Avg), which showed no statistically significant differences (*p* > 0.407).

Tables  1(a) and 1(b) show the values of the enamel thickness after the completion of orthodontic treatment depending on the applied adhesive system.

## 4. Discussion 

The presented results show that the enamel thickness after completed treatment and its possible damage is not dependent in any way on the type of adhesive system. The studies by other authors, cited above, suggest a smaller impact of the self-etching system on the enamel and the performed experiment leads to the conclusion that the impact of the two systems on the enamel is similar. The methodology of the compared research is different. Our study focused on the quantitative assessment of the enamel, while the previously mentioned experiments by other authors, Retief [1], Arakawa et al. [2], Asmussen [3], and Charbeneau Voss and Charbeneau [4], assessed the enamel quality. They measured the amount of dissociated calcium and depth of penetration of resin hooks. Therefore, it can be concluded that the results of the compared studies are not contradictory, since they measure different characteristics of the enamel. The use of an etchant does not reduce the enamel thickness due to the lack of abrasive abilities. The method of etching can only indirectly influence the final tissue thickness by substantial weakening of its structure, which increases the enamel sensitivity to operator intervention during debonding and cleaning. So far, the evaluation of the full tissue thickness has been difficult to carry out, so there are not many publications to refer to when discussing the results. Accordingly, in order to expand the available knowledge on this topic and objectify it, an attempt was made to use the OCT to assess the quality of the enamel after using various types of adhesive systems. A new application of the above-mentioned device was to evaluate the diversity of the image depending on the size and depth of the generated pores of the enamel, which affect the propagation of light waves in the tissue and the appropriate image registration. The result obtained has led to the conclusion that the use of self-etching systems is safe for the enamel.

Many independent studies describe the features of self-etching systems, which include small aggressiveness in relation to enamel. They result in substantially lower, than in the case of classic etching, irreversible changes in the tissue and affect the production of shorter resin hooks. However, they generate a sufficient bonding strength for the clinical procedure and there are rarer cases of bonding errors in the enamel-adhesive system phase than the classic etching method [26–28]. A significantly greater bonding strength of the self-etching system was confirmed in the studies of Bishara et al. and Buyukyilmaz et al. [17, 29]. These studies challenged the hypothesis of many critics such as Fjeld and Øgaard [30] and research groups led by the previously cited Bishara et al. [30–35], who hypothesized greater risks of self-etching systems in their experiments. It was associated with increased adhesion errors in the enamel-adhesive system phase. These errors increased the risk of cracks in the enamel. In this context, the performed studies have proven the superiority of the self-etching system over the classic one.

Such significant differences in assessing the strength of the adhesive system between many researchers may be related to the quality and type of selected test samples. Published studies were performed on extracted human or animal teeth, both front and back ones. Diversity of observations may be related to the method of testing, both in vitro and in vivo, as well as the preparation of the sample surface, the use of various orthodontic adhesive materials, debonding methods, the time after which the hooks were removed, and the conditions of storing samples.

The presented OCT method can be compared to other methods of imaging of the enamel layer. The known methods of tooth enamel analysis include assessment by means of an atomic force microscope (AFM) [36, 37] and a scanning electron microscope (SEM) [38].

The other known methods of enamel thickness analysis do not enable automatic, quantitative measurement of the enamel thickness present in the ROI and automatic comparison of image groups. This is the case in [39], where comparisons between specific areas of the tooth enamel were made manually in OCT images. Automatic measurement was presented only in [40]. However, it concerns polarization sensitive optical coherence tomography (PS-OCT) and is not related to the problem of overlap of individual images in the subsequent processing stages of the tooth as shown in this paper.

## 5. Conclusions

The range of variations in the enamel thickness after treatment with fixed thin-arched braces are not subject to modification of a factor such as the type of adhesive system.

The OCT is an effective diagnostic tool to evaluate the thickness of the enamel tissue before and after the completed orthodontic treatment.

## Figures and Tables

**(a):** 

Variables	*n*	M	Me	Min.	Max.	Q1	Q3	*R*	SD
*V*_Avg	40	472,75	453,60	259,16	743,87	376,28	564,13	187,85	117,25
*V*_Min	40	128,18	130,00	0,00	360,00	80,00	185,00	105,00	78,36
*V*_Max	40	1093,62	1040,00	450,00	2755,00	845,00	1255,00	410,00	411,96
Dif_Avg	40	96,53	76,50	−142,77	563,00	30,68	160,11	129,43	104,91
Dif_Min	40	55,71	55,00	−50,00	180,00	20,00	85,00	65,00	46,53
Dif_Max	40	432,69	180,00	−915,00	3060,00	15,00	700,00	685,00	665,75

The following symbols have been used in the table: *n*, number of samples; M, arithmetic mean; Me, median; Min–max, range of variation; Q1–Q3, first quartile, third quartile; *R*, interquartile range; SD, standard deviation; *V*_Avg, average enamel thickness prior to orthodontic treatment; *V*_Min, minimum enamel thickness prior to orthodontic treatment; *V*_Max, maximum enamel thickness prior to orthodontic treatment; Dif_Avg, difference in average enamel thickness prior to orthodontic treatment and after its completion; Dif_Min, difference in minimum enamel thickness prior to orthodontic treatment and after its completion; Dif_Max, difference in maximum enamel thickness prior to orthodontic treatment and after its completion.

**(b):** 

Variables	*n*	M	Me	Min.	Max.	Q1	Q3	*R*	SD
*V*_Avg	40	469,03	439,72	172,14	844,79	367,49	570,38	202,95	130,18
*V*_Min	40	132,26	140,00	0,00	315,00	80,00	185,00	95,00	69,89
*V*_Max	40	1103,84	1030,00	460,00	2515,00	805,00	1330,00	400,00	432,52
Dif_Avg	40	90,93	65,15	−65,84	461,71	23,35	142,68	120,49	96,19
Dif_Min	40	50,15	40,00	−185,00	220,00	10,00	90,00	75,00	52,61
Dif_Max	40	402,10	265,00	−1415,00	3215,00	35,00	685,00	630,00	569,47

The following symbols have been used in the table: *n*, number of samples; M, arithmetic mean; Me, median; Min–max, range of variation; Q1–Q3, first quartile, third quartile; *R*, interquartile range; SD, standard deviation; *V*_Avg, average enamel thickness after orthodontic treatment; *V*_Min, minimum enamel thickness after orthodontic treatment; *V*_Max, maximum enamel thickness after orthodontic treatment; Dif_Avg, difference in average enamel thickness prior to orthodontic treatment and after its completion; Dif_Min, difference in minimum enamel thickness prior to orthodontic treatment and after its completion; Dif_Max, difference in maximum enamel thickness prior to orthodontic treatment and after its completion.
