# Corrigendum to “Enamel Thickness before and after Orthodontic Treatment Analysed in Optical Coherence Tomography”

**DOI:** 10.1155/2020/7291314

**Published:** 2020-05-14

**Authors:** Monika Machoy, Julia Seeliger, Robert Koprowski, Krzysztof Safranow, Tomasz Gedrange, Krzysztof Woźniak

**Affiliations:** ^1^Division of Orthodontics, Pomeranian Medical University, Szczecin, Poland; ^2^Division of Orthodontics, Technical University Dresden, Dresden, Germany; ^3^Department of Biomedical Computer Systems, University of Silesia, Faculty of Computer Science and Materials Science, Institute of Computer Science, Katowice, Poland; ^4^Department of Biochemistry and Medical Chemistry, Pomeranian Medical University, Szczecin, Poland

In the article titled “Enamel Thickness before and after Orthodontic Treatment Analysed in Optical Coherence Tomography” [1], the order of the authors was modified. The revised authors' list and affiliations are shown above.
